# Combined Toxic Effects of Heavy Metals and Antibiotics on a *Pseudomonas fluorescens* Strain ZY2 Isolated from Swine Wastewater

**DOI:** 10.3390/ijms16022839

**Published:** 2015-01-27

**Authors:** Yan Zhou, Yan-Bin Xu, Jia-Xin Xu, Xiao-Hua Zhang, Shi-Hui Xu, Qing-Ping Du

**Affiliations:** School of Environmental Science and Engineering, Guangdong University of Technology, Guangzhou 510006, China; E-Mails: hjzhouyan@163.com (Y.Z.); touchxjx@163.com (J.-X.X.); zhxhuaxh@163.com (X.-H.Z.); crystalxss@163.com (S.-H.X.); duqingping@gdut.edu.cn (Q.-P.D.)

**Keywords:** *Pseudomonas fluorescens*, resistance, antibiotic, heavy metal, swine wastewater

## Abstract

A *Pseudomonas fluorescens* strain ZY2, isolated from swine wastewater, was used to investigate the synergistic effects of five heavy metals (Pb, Cu, Zn, Cr(VI) and Hg) on bacterial resistance to antibiotics. Results indicate that the combined effects of antibiotic type, heavy metal type and concentration were significant (*p* < 0.01). Cross-resistance to Hg and antibiotics was the most noticeable. Moreover, the resistance to Hg and cefradine or amoxicillin, and Cr and amoxicillin were synergistic for low heavy metal concentrations, and turned antagonistic with increasing concentrations, while the resistances to Cr or Cu and cefradine, Pb or Cu and amoxicillin, Cu and norfloxacin showed reverse effects. In addition, resistance to Zn and amoxicillin were always synergetic, while resistance to Pb and cefradine or norfloxacin, Cr or Hg and norfloxacin as well as all the heavy metals and tetracycline were antagonistic. These results indicate that bacterial resistance to antibiotics can be affected by the type and concentration of co-exposed heavy metals and may further threaten people’s health and ecological security severely via horizontal gene transfer.

## 1. Introduction

Antibiotics and heavy metals are two common types of typical environmental pollutants from effluent and industrial activities, and both are hazardous to public health and ecological safety [1]. Though their individual effect on test organisms and the environment has been studied for more than thirty years [2,3], very little information is available on their combined effects.

Bacterial resistance to heavy metals and antibiotics can be developed after a low-level and prolonged exposure to these two types of common environmental pollutants [4]. Mechanisms of efflux pumps, spontaneous chromosomal mutations and conjugative plasmids are believed to be the general bacterial resistance mechanisms against antibiotics, which can also explain the bacterial co-resistance to both antibiotics and metals in ecosystem [5,6,7]. It is known that the bacterial antibiotic resistance genes (ARGs) are genetic elements, such as plasmids, and can be expressed through inductive process, persist and spread via horizontal gene transfer, even in the absence of antibiotics [8,9,10]. Once antibiotic resistance genes are acquired by pathogens or environmental microorganisms, they may severely threaten human health and ecological security [11].

Environmental problems associated with heavy metals are a direct result of industrial activities such as the extraction of minerals or metals and the discharges [12]. Metals are subjected to transformation and detoxification through biological processes [13,14,15] and/or chemical ones [16,17,18]. To removal metals from ecosystem, especially sediments and soils, phytoremediation is preferred to microbiological ones as the former can completely accumulate and move the metals from the matrices [19,20,21,22]. Because of the wide occurrence of metals in the environment, they can serve as typical stress factors and may also be antibiotic resistance determinants when both of them occur simultaneously [23,24]. It is very important to elucidate the bacterial resistance to both heavy metals and antibiotics in detail for further understanding the bacterial cross-resistance and its ecological risk. In the present paper, we studied the resistances of *Pseudomonas fluorescens* ZY2 to four antibiotics (cefradine, norfloxacin, amoxicillin and tetracycline) and five heavy metals (Pb, Cu, Zn, Cr and Hg) used with different concentrations. The results will be helpful in further revealing the ecological harm and human health hazard of complex pollutants.

## 2. Results and Discussion

### 2.1. Heavy Metal Resistance and Antibiotic Resistance

*Pseudomonas* sp*.* are widely found in pristine and contaminated soils or waters and are known for their resistance to a wide range of stressors including chemicals [25], especially *P. fluorescens* [26]. In the present paper, a main isolate from swine wastewater, identified as *P. fluorescens* ZY2, was chosen to investigate its resistance to some metals and antibiotics which were frequently related to human activities or additives in livestock feed. The bacterial resistance to heavy metals was determined using the concentration gradient design and the recorded bacterial minimum inhibitory concentrations (MICs) of Pb, Cu, Zn, Cr and Hg were 125, 100, 100, 100 and 25 mg/L, respectively. The biological toxicity of Hg was the largest and that of Pb was the smallest, which were different from that of Cu > Cd > Mn > Zn > Cr > Pb reported by Matyar *et al*. [27], and might be considered closely related to the bacterial habitat. However, the strong toxicity of Hg has been widely recognized [28]. Therefore, the tested concentrations of Pb (0, 0.1, 2, 10, 50 and 100 mg/L), Cu (0, 0.1, 2, 10, 50, 100 mg/L), Zn (0, 0.1, 2, 10, 50, 100 mg/L), Cr (0, 0.1, 2, 20, 50, 80 mg/L) and Hg (0, 0.1, 1, 5, 10, 20 mg/L) in the culture media were pre-imposed to determine bacterial antibiotic resistance.

Nine parallel tests were conducted to determine antibiotic resistance. All results of quality control strains meet the requirements of Clinical and Laboratory Standards Institute (CLSI) [29]. Based on the means of zone diameter measurements using a standard caliper, the bacterial resistance to these antibiotics is in a descending order as tetracycline, amoxicillin, norfloxacin, and cefradine. Moreover, *P. fluorescens* ZY2 showed resistance to amoxicillin and tetracycline, and intermediate resistance to cefradine and norfloxacin with reference of the criterion from the document M100-S22 [29] (Table 1). Because bacterial resistance to stress factors is strongly dependent on an indigenous genetic basis and biological capability [26], *Pseudomonas* spp*.* from Iskenderun Bay showed both the strongest and weakest resistance to ampicillin and cefepime, respectively [28]. It was suggested that both tetracycline and amoxicillin were common antibiotics widely used in specialized pig-farms [1]. Similarly, selective heavy metals were also used in pig feed and released through pig manure into the environment [28].

**Table 1:** Susceptibility of *P. fluorescens* ZY2 to four kinds of antibiotics.

Antibiotic (µg/disc)	CLSI Category			*P. fluorescens* ZY2	
	Sensitive (mm)	Intermediary (mm)	Resistant (mm)	Mean of Inhibitory Zones (mm)	Sensitive Case
Cefradine (30)	≥18	15~17	≤14	15.28 ± 0.26	Intermediate
Norfloxacin (10)	≥17	13~16	≤12	14.62 ± 0.19	Intermediate
Amoxicillin (10)	≥18	14~17	≤13	12.70 ± 0.63	Resistant
Tetracycline (30)	≥15	12~14	≤11	0.00 ± 0.00	Resistant

### 2.2. Results of the Binary Exposure

Bacterial isolates from the contaminated soils [30], surface water [31] and even the Antarctic shallow sediments [32], have been reported to have cross-resistance to both heavy metal and antibiotic, while the detailed mechanisms of the cross-resistance are still unclear.

**Figure 1:**
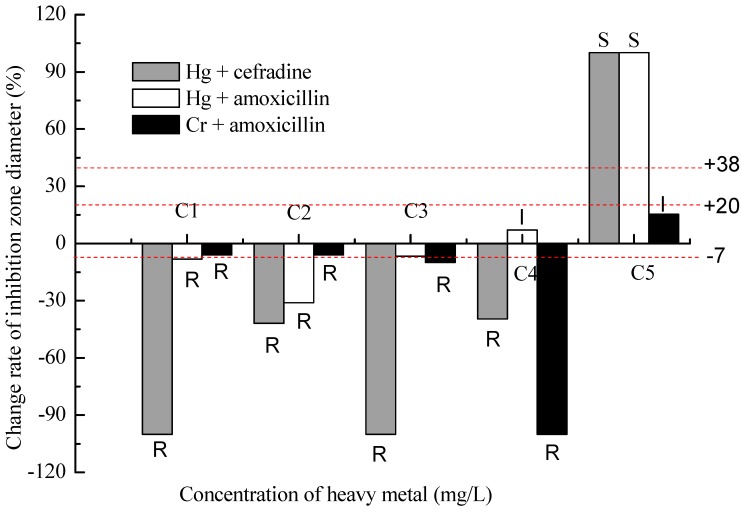
Effect of heavy metals on antibiotic resistance turning from synergistic to antagonistic with their increasing concentration. Each value in Figure 1 is the mean of nine replicates. R (Resistant), I (Intermediate) and S (Sensitive) of the CLSI category were labeled on each column. C1, C2, C3, C4, C5 were the effective concentrations of heavy metal in the medium, that is, Cr^6+^ of 0.06, 1.88, 19.18, 46.70, 77.59 mg/L, and Hg^2+^ of 0.07, 0.86, 4.96, 9.24, 19.38 mg/L, respectively. For the convenience of description and comparison, the design concentrations of heavy metals were still used in the following subsequent discussions. The red lines are the critical values of change rates which are counted according to the CLSI category showing in Table 1 and the symbols of “R”, “I” or “S” are also the results of the comparison between the change rate and the critical value of the same antibiotic.

**Figure 2:**
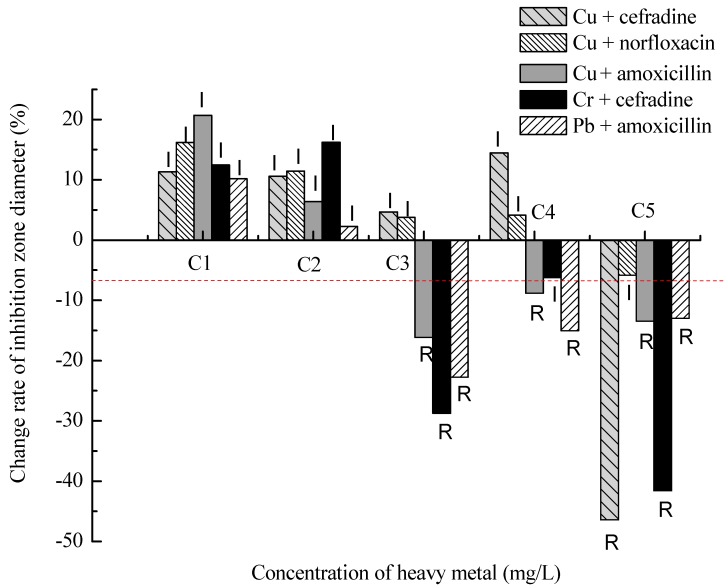
Effect of heavy metals on antibiotic resistance turning from antagonistic to synergistic with their concentrations increasing. R (Resistant), I (Intermediate) and S (Sensitive) of the CLSI category were labeled on each column. C1, C2, C3, C4, C5 were the effective concentrations of heavy metal in the medium, that is, Pb^2+^ of 0.06, 1.66, 9.25, 49.37, 98.02 mg/L, Cu^2+^ of 0.07, 1.66, 9.96, 46.17, 98.68 mg/L, and Cr^6+^ of 0.06, 1.88, 19.18, 46.70, 77.59 mg/L, respectively. The red lines are the critical values of change rates which are counted according to the CLSI category shown in Table 1, and the symbols of “R”, “I” or “S” are the results of the comparison between the change rate and the critical value of the same antibiotic.

**Figure 3:**
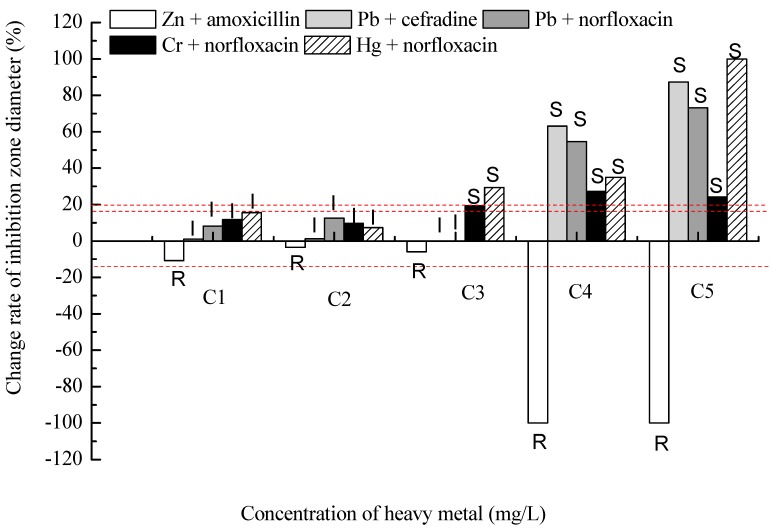
Effect of heavy metals on antibiotic resistance unchanged with heavy metal concentration. Some histograms cannot be seen since the inhibition zone size of some antibiotics are unaffected by certain concentrations of some heavy metals. R (Resistant), I (Intermediate) and S (Sensitive) of the CLSI category were labeled on each column. C1, C2, C3, C4, C5 were the effective concentrations of each heavy metal in the medium, that is, Pb^2+^ of 0.06, 1.66, 9.25, 49.37, 98.02 mg/L, Cu^2+^ of 0.07, 1.66, 9.96, 46.17, 98.68 mg/L, Zn^2+^ of 0.07, 1.78, 8.97, 44.96, 90.34 mg/L, Cr^6+^ of 0.06, 1.88, 19.18, 46.70, 77.59 mg/L, and Hg^2+^ of 0.07, 0.86, 4.96, 9.24, 19.38 mg/L, respectively. The red lines are the critical values of change rates, which are counted according to the CLSI category shown in Table 1, and the symbols of “R”, “I” or “S” are also the results of the comparison between the change rate and the critical value of the same antibiotic.

According to the statistical evaluation of results conducted with SPSS Version 17.0, it was found that the comprehensive effect of antibiotic type, heavy metal type and heavy metal concentration on inhibitory zone size of *P. fluorescens* was significant (*p* < 0.01). Therefore, both the type and concentration of heavy metals affected the bacterial resistance to antibiotic. For *P. fluorescens* ZY2, the influence of heavy metal on resistance to antibiotic was also different from type to type, and the influence of Hg was the most significant (Figure 1, Figure 2 and Figure 3). Moreover, Hg was the only heavy metal of which the MIC decreased with the addition of antibiotic discs onto the media, for example, tetracycline decreased the MIC of Hg from 25 to 10 mg/L (data not shown), which was supported by its special physicochemical properties and extreme toxicity [33]. Besides, *P. fluorescens* ZY2 was very sensitive to Hg concentration and exposure. The change rates of inhibitory zone size varied from −100% to 100% with increasing Hg concentration. The bacterial resistance to tetracycline was the strongest, but decreased with the increase in Hg until the bacterial resistance to tetracycline was lost at 10 mg/L of Hg. Similarly, Singh *et al.* [34] found that *P. fluorescens* is resistant only at very low concentrations of Hg.

Most studies have focused on the effect of heavy metal type on the bacterial antibiotic resistance [35]. For instance, Cu and Zn could increase antibiotic resistance in the porcine microflora while Hg could decrease the antibiotic resistance [28]. However, the concentration-dependent effect of heavy metals on bacterial antibiotics resistance has been generally ignored.

There was a positive correlation between low heavy metal concentrations and bacterial antibiotic resistance, however, the correlation turned to a negative one with increasing concentration of the heavy metal (Figure 1). For example, bacterial resistance to cefradine was strongly pronounced at low Hg concentrations (less than 10 mg/L), and only high Hg concentration (design concentration of 20 mg/L) resulted in a decrease in bacterial resistance to cefradine. The effects of Hg and Cr on the bacterial resistance to amoxicillin were similar, but the transitional concentrations were 10 and 80 mg/L for Hg and Cr, respectively. Hg of 20 mg/L could change the bacterial susceptibility to both cefradine and amoxicillin from resistance to sensitivity.

On the contrary, the bacterial antibiotic resistance was negatively related to some heavy metals at low concentrations, whereas it was positively related to them at high concentrations, such as between Cr and cefradine, Pb and amoxicillin, Cu and cefradine, Cu and norfloxacin, Cu and amoxicillin. The bacterial resistance to amoxicillin increased obviously with co-exposure of 10 mg/L Cu or Pb as well as the binary exposure of cefradine and 20 mg/L Cr or 100 mg/L Cu (Figure 2).

**Figure 4:**
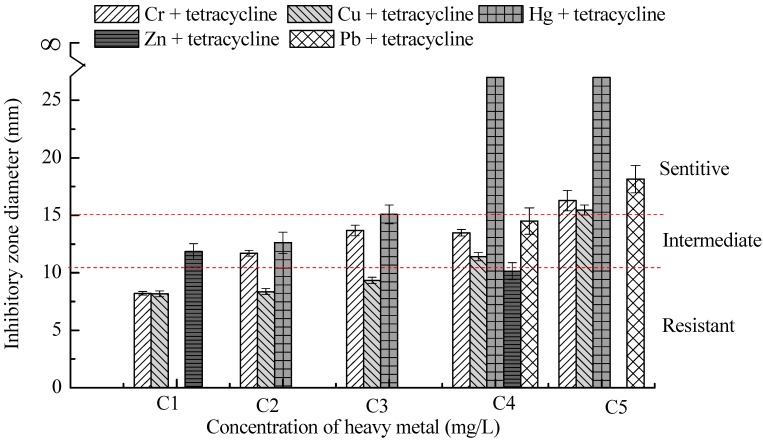
Effect of heavy metals on tetracycline resistance. Some histograms are not shown because their inhibition zone diameters were zero. Resistant, Intermediate and Sensitive were labeled according to the critical values of tetracycline in Table 1. C1, C2, C3, C4, C5 were the effective concentrations of each heavy metal in the medium, that is, Pb^2+^ of 0.06, 1.66, 9.25, 49.37, 98.02 mg/L, Cu^2+^ of 0.07, 1.66, 9.96, 46.17, 98.68 mg/L, Zn^2+^ of 0.07, 1.78, 8.97, 44.96, 90.34 mg/L, Cr^6+^ of 0.06, 1.88, 19.18, 46.70, 77.59 mg/L, and Hg^2+^ of 0.07, 0.86, 4.96, 9.24, 19.38 mg/L, respectively.

Further, bacterial resistance to some antibiotics had no relationship with the concentrations of heavy metals (Figure 3). For example, there was an obvious synergetic effect between resistance to Zn and amoxicillin, and an obvious antagonistic effect between resistance to Pb and cefradine, as well as Pb and norfloxacin, Cr and norfloxacin or Hg and norfloxacin. In addition, the strain was strongly resistant to tetracycline and the inhibition zone diameter remained zero, though all heavy metals of certain concentrations could increase the inhibition zone diameter, and the susceptibility of *P. fluorescens* ZY2 to tetracycline started to divide into three types: 48% resistant, 32% intermediary and 20% sensitive (Figure 4). The susceptibility of *P. fluorescens* ZY2 to tetracycline turned out to be sensitive when the concentrations of Hg, Pb, Cr and Cu were more than 5, 100, 80 and 100 mg/L, respectively, while the effect of Zn on bacterial susceptibility to tetracycline was minimal, which was about the same as the toxicity order of the heavy metals. Therefore, the heavy metal resistance and the antibiotic resistance might be either synergistic or antagonistic, which depended on the specific type and concentration of the heavy metal. Moreover, we should pay more attention to the phenomenon that bacterial resistance to antibiotics could be enhanced by some heavy metals of certain concentrations.

In the present study, the complex results were dependent on the types of heavy metal and antibiotic, as well as the concentration of heavy metal, which might be explained from two aspects, the chemical reactions between heavy metal and antibiotic and their individual bio-effect. The effective concentrations of heavy metals or antibiotics may be affected by the chemical reactions between them [36]. If the products from heavy metal-antibiotic reaction are more toxic than the parent substances, the bacterial resistance to the antibiotic might be weakened [37]. However，when the toxicity of the products is no more than that of the parent substances, the bacterial antibiotic resistance can be unchanged or strengthened for the effective decreasing concentration. For instance, the presence of Zn or Cu can increase resistance to imipenem in *P. aeruginosa* because of possible coagulation [38]. Thus, it is conceivable that chemical reactions can be affected by the type and concentration of heavy metals, antibiotics and even other pollutants present in the same system.

In addition, the effect of heavy metal on bacterial resistance to antibiotics might also lead to the phenomenon of bacterial cross-resistance, and the relative mechanism might be very complex. Generally, as a key substance of protein and enzyme activity, heavy metals in trace amounts are essential to bacterial growth [39], but could be stress factors affecting the synthesis of proteins in *P. fluorescens* [40]. Moreover, when some efflux pumps and integron-containing mobile elements responsible for the cross-resistance between heavy metals and antibiotics are affected by some stress factors [41], the cross-resistance also can be impaired, which might explain the synergetic resistance to both Cr and some antibiotics. Some cryptic plasmids in Vibrio species may be ecologically important to their resistance to Hg and antibiotics [6,42]. Moreover, low heavy metal concentrations can induce expression of various proteins, especially metallothioneins, and the synthesis of metallothioneins has often been shown to be prompted by elevated concentrations of some metals and might further inhibit antibiotic resistance instead of enhancing it [43].

Though the heavy metal stress on bacterial resistance to antibiotics has recently been recognized, the exact mechanisms remain unclear because of the lack of sufficient studies on simultaneous resistance to specific heavy metals and antibiotics in microbial species. Study on the cross-resistance for a specific bacterium would be helpful to reveal the environmental behavior and risk of heavy metals and antibiotics.

## 3. Experimental Section

### 3.1. Characteristics of the Strain

The bacterial strain used in this study was isolated from the swine wastewater of a livestock farm in Guangzhou, China, by the method of ten-fold serial dilutions and plating on Luria-Bertani (LB) medium agar plates incubated at 30 °C. Colonies developed on agar plates were streaked on fresh agar plates for purification until pure culture was established. The pure culture of isolates was further identified as *Pseudomonas fluorescens* ZY2 (EU854430) (see Figure S1) by a combination of the morphological, biochemical and 16S rRNA sequence analysis.

### 3.2. Determination of the Resistance to Heavy Metals

The bacterial active culture was prepared by inoculating *P. fluorescens* ZY2 from agar plates into sterilized LB liquid medium and incubating at 30 °C for 16 h. To achieve an adequate inoculum, the density of the bacterial suspension was diluted to about 10^8^ cfu/mL by sterile saline. And the MICs testing was conducted on the Mueller–Hinton agar mediums containing Pb, Cr, Hg, Cu or Zn with the concentration ranging from 0 to 400 mg/L. The effective concentration of heavy metals in the medium was determined by atomic absorption spectrophotometer (Hitachi, Z2000, Tokyo, Japan) for Cu^2+^, Zn^2+^ and Pb^2+^ according to the national standard method (GB 7475-87), a cold vapor atomic fluorescence spectrometry (AF-640, Beijing Rayleigh Analytical Instrument Corporation, Beijing, China) for Hg^2+^ according to the national standard method (HJ 597-2011) and diphenylcarbonyl-2-hydrazine spectrophotometric method for Cr^6+^ according to the national standard method (GB 7467-87) [44]. During the testing, the control strain was *Escherichia coli* K-12*.*

### 3.3. Antimicrobial Susceptibility Testing

The susceptibilities of *P. fluorescens* ZY2 to antimicrobials were determined by the standard Kirby–Bauer disc-diffusion method for the antibiotic discs of cefradine (30 μg), norfloxacin (10 μg), amoxicillin (10 µg) and tetracycline (30 μg) according to the Clinical and Laboratory Standards Institute guidelines [29]. The disk diffusion Mueller–Hinton agar plates were measured manually using a standard caliper. The means of zone diameter measurements were calculated from nine independent readings using antibiotic disk diffusion inhibition zones of *Pseudomonas aeruginosa* ATCC 27853 (quality control strain).

### 3.4. Binary Exposure Experiment

Based on the results of the MIC tests, Mueller–Hinton agar plates with any of these five heavy metals were prepared and 0.1 mL of the bacterial suspension was then spread on them. And four antibiotic discs were placed on the inoculated media after 10 min, respectively, according to the experimental design. After a culturing period for 24 h at 37 °C, the relationship between the antibiotics and heavy metal resistance was analyzed based on the values of the inhibitory zone diameters measured by a Vernier caliper (±0.001 mm), combined with bacterial growth. *Escherichia coli* ATCC 25922, *Escherichia coli* ATCC 35218 and *Pseudomonas aeruginosa* ATCC 27853 were used for quality controls.

Nine parallel tests were conducted to avoid error.

### 3.5. Statistics

Statistical evaluation of the data was conducted with SPSS Version 17.0 (Statistical Package for the Social Sciences, IBM Corp, Chicago, IL, USA). Analysis of variance was performed with inhibitory zone size as the dependent variable and heavy metal concentration, antibiotic type and heavy metal type as fixed factors. The level of significance was accepted as *p* < 0.05.

## 4. Conclusions

*P. fluorescens* ZY2, an isolate from a pig farm, was resistant to several heavy metals and antibiotics. Moreover, the strongest and weakest resistance was for Cu and Hg, respectively. The analysis of variance indicated that the individual effects of antibiotic, heavy metal and concentration were significant (*p* < 0.05). It was observed that the impact of heavy metals on bacterial antibiotic resistance was positive or negative according to the types and concentrations of heavy metals and antibiotics present. Heavy metals can affect the antibiotic resistance in environmental microorganisms. Co-existence of antibiotic and heavy metal can alter their individual effects on the extent of pollution, as well as on biological removal of pollutants, which should be taken into account during the bioremediation processes and toxicological evaluation.

## Supplementary Materials

Supplementary materials can be found at http://www.mdpi.com/1422-0067/16/02/2839/s1.
